# Auditory frequency discrimination in developmental dyslexia: A meta‐analysis

**DOI:** 10.1002/dys.1645

**Published:** 2019-12-26

**Authors:** Caroline Witton, Katy Swoboda, Laura R. Shapiro, Joel B. Talcott

**Affiliations:** ^1^ Aston Neuroscience Institute Aston University Birmingham UK

**Keywords:** auditory, developmental dyslexia, frequency discrimination, meta‐analysis, phonological awareness, reading

## Abstract

Auditory frequency discrimination has been used as an index of sensory processing in developmental language disorders such as dyslexia, where group differences have often been interpreted as evidence for a basic deficit in auditory processing that underpins and constrains individual variability in the development of phonological skills. Here, we conducted a meta‐analysis to evaluate the cumulative evidence for group differences in frequency discrimination and to explore the impact of some potential moderator variables that could contribute to variability in effect‐size estimations across studies. Our analyses revealed mean effect sizes for group differences on frequency discrimination tasks on the order of three‐quarters of a standard deviation, but in the presence of substantial inter‐study variability in their magnitude. Moderator variable analyses indicated that factors related both to participant variability on behavioural and cognitive variables associated with the dyslexia phenotype, and to variability in the task design, contributed to differences in the magnitude of effect size across studies. The apparently complex pattern of results was compounded by the lack of concurrent, standardised metrics of cognitive and reading component skills across the constituent studies. Differences on sensory processing tasks are often reported in studies of developmental disorders, but these need to be more carefully interpreted in the context of non‐sensory factors, which may explain significant inter‐ and intra‐group variance in the dependent measure of interest.

## 1 INTRODUCTION

Developmental dyslexia manifests as an impairment of the normal developmental trajectory of reading‐skill acquisition, for which difficulties in phonological skills are a predominant, proximal cause (Stanovich, 1988). In a broad sense, phonological skills can be defined as an ability to attend to and process the segmental structure of language. Speech is a complex auditory signal, containing dynamic changes in pitch and amplitude that require accurate detection, often in the presence of background noise. At a basic level, therefore, robust auditory processing is one of a number of theoretical pre‐requisites for the development of competent phonological skills. Atypical auditory processing has been reported in dyslexia for a wide range of acoustic stimuli (see Farmer & Klein, 1993; Habib, 2000; Hämäläinen, Salminen, & Leppänen, 2013, for review), but the nature and significance of these deficits to aetiological models of reading impairments remain under‐defined. For example, does the presence of deficits reflect a basic‐sensory level factor relevant to the causal aetiology of dyslexia, evidence for a more generalised difference in brain function, or do they manifest from methodological artefacts of the sampling or measurement techniques employed in a given study?

One of the more frequently‐studied aspects of auditory processing in developmental dyslexia is pitch perception, conventionally measured through tasks of frequency discrimination – in their quantitative review, Hämäläinen et al. (2013) reported an average effect‐size of 0.7 for group differences across 22 studies.^1^ In this study, we have exploited this relatively extensive literature on frequency discrimination in dyslexia to conduct a more detailed meta‐analysis of how the magnitude of effect‐sizes obtained in such auditory processing tasks may be moderated by reading, non‐reading cognitive variables, or to effects associated with the design of the task. Through adopting this meta‐analytic approach, we aimed to harness the increased statistical power that results from pooling data across studies, and explore the sources through which potential heterogeneity in effect‐size estimates may arise.

## 2 POTENTIAL SOURCES OF HETEROGENEITY IN AUDITORY PROCESSING TASK PERFORMANCE

Performance in psychophysical tasks always depends, to some extent, on the cognitive skills of the listener – including the memory and attention skills required within and across large numbers of experimental trials – as well as their sensory sensitivity. The load on non‐sensory cognitive skills in such tasks may be particularly high in non‐expert listeners and in children who have less experience in performing these types of behavioural task. Threshold for frequency discrimination is usually estimated from psychophysical paradigms consisting of a long series of individual trials, each of which requires that the participant listens to a sequence of two or more tones and make judgements about their relative pitch (for e.g., whether one was “higher,” or whether the tones presented in a pair were the “same” or “different”). In studies of developmental disorders – where these tasks have become commonplace – threshold has been typically obtained using an adaptive procedure, in which the frequency‐difference is adjusted on a trial‐by‐trial basis, according to the participant's previous response pattern. Expert listeners are able to discriminate pitch‐contrasts on the order of 0.1–0.3% in the frequency range around 1 kHz, that is, approximately 1–3 Hz (e.g., Henning, 1970; Moore, 1973; Nordmark, 1968; Rosenblith & Stevens, 1953; Wier, Jesteadt, & Green, 1977). However, in studies of developmental disorders such as dyslexia, reported thresholds are often substantially higher than in these “traditional” psychophysical studies, suggesting that variables other than sensory sensitivity influence the threshold measurement (Halliday & Bishop, 2006; Heath, Bishop, Hogben, & Roach, 2006).

It follows, therefore, that the reliability and validity of behavioural measurements in studies of frequency discrimination could be influenced by individual differences in non‐sensory, cognitive skills between and within groups of participants (Witton, Talcott, & Henning, 2017). For example, developmental dyslexia has been associated with variability in short term and working memory (Jeffries & Everatt, 2004; Wang & Gathercole, 2013). Correspondingly, psychometric measures of digit span which load onto short term memory constructs have been revealed previously as significant predictors of variance in thresholds, both for frequency discrimination (Banai & Ahissar, 2004, 2006) and for other auditory psychophysical tasks (Hulslander et al., 2004; Witton, Stein, Stoodley, Rosner, & Talcott, 2002). Dyslexia also has high levels of diagnostic co‐occurrence with attention‐deficit hyperactivity disorder (ADHD), where from 25 to 40% of children with either dyslexia or ADHD meet the diagnostic criteria for the other (Wilcutt & Pennington, 2000). Because some attention skills also co‐vary with psychophysical task performance, both in children (Moore, Ferguson, Edmondson‐Jones, Ratib, & Riley, 2010; Moore, Ferguson, Halliday, & Riley, 2008; Talcott et al., 2002; Talcott, Witton, & Stein, 2013), adults (Zhang, Barry, Moore, & Amitay, 2012), and in simulations of non‐expert listeners (Witton et al., 2017), it is likely that the frequency discrimination impairments reported in the literature were also influenced to some degree by factors other than the reading skills that primarily distinguish the dyslexic listeners from the control groups. Despite this, few studies of frequency discrimination in dyslexia have sufficiently measured attention skills, or consistently screened for ADHD symptoms, in their participants. Different types of task design may tap into memory capacity, attention skills or more generalised constructs such as non‐verbal IQ to variable extent. For example, differences in the task's requirement for the subject to retain memory traces of the stimuli, to verbalise concepts such as pitch (i.e., “higher” and “lower”), or the number of times a target stimulus is repeated, are all factors which alter the cognitive demands of the task and could contribute to variability in threshold estimates at an individual level. Variability in task demands across studies could therefore alter thresholds via their impact on individual differences in general or domain specific cognitive skills, without necessarily reflecting any direct statistical relationship with reading ability. One aim of the analyses reported here, therefore, is to determine whether the literature on frequency discrimination in dyslexia can provide any evidence for such third variable effects, both through meta‐regressions with the limited measures of cognitive skill that are available in this literature, and through an analysis of effect‐sizes across different psychophysical task‐designs.

The inherent heterogeneity in the dyslexia phenotype (Snowling, 2008; Talcott et al., 2013), which manifests in significant intra‐group variability across behavioural and cognitive processing variables, compounds the difficulty in interpreting psychophysical data, because cognitive skills (which may often be uncontrolled in sampling within quasi‐experimental designs) will impact to variable extent, depending upon the demands induced by different psychophysical tasks. For example, a task which provides more than one exemplar of a control stimulus on any given trial may load upon short term memory differently than a task which presents each stimulus only once. The potential influence of such cognitive third variables on the relationship between sensory thresholds and reading is also illustrated by the observation that, despite seemingly robust group differences in frequency discrimination, correlations with reading sub‐skills such as in phonological decoding are generally found to be relatively modest (Ahissar, Protopapas, Reid, & Merzenich, 2000; Talcott et al., 2002). Similar patterns have been reported for visual tasks in dyslexia measured with psychophysical methods, that is, stronger effects occur at the group level in the presence of comparatively lower magnitudes of association with measures of reading skill (Benassi, Simonelli, Giovagnoli, & Bolzani, 2010). This evidence supports the view that sensory impairments may (also) be associated with other behavioural variables, which may be neither carefully measured nor controlled in quasi‐experimental research designs (Witton & Talcott, 2018). In summary, it is likely that at least some of the variability between studies on sensory processing tasks results from differences in sample selection or ascertainment (McArthur & Bishop, 2004a), in which the presence of uncontrolled cognitive variables (McArthur & Bishop, 2004b; Roach, Edwards, & Hogben, 2004) or developmental factors (Owens, Dawes, & Bishop, 2008) contribute both to high inter‐individual variability across studies (McArthur & Hogben, 2012) and to associated differences in effect‐sizes across groups (Talcott et al., 2013).

The meta‐analysis and *post hoc* moderator variable analyses presented here were motivated by the aims to better quantify the consistency to which group differences in frequency discrimination are associated with reading disability, and to investigate how both participant‐ and experimental‐ factors may explain the variability in effect‐sizes across studies. Improved understanding of the impact of these factors will help elucidate the extent to which frequency discrimination is directly associated with disorder phenotypes, or alternatively related to the presence of third variables, such as study‐specific factors, either in task design or in participant sampling.

## 3 METHODS

### 3.1 Selection criteria and data collection

We aimed to identify the population of studies which obtained psychophysical measurements of frequency discrimination thresholds in participants with diagnoses of developmental dyslexia, compared to a control group. Relevant studies were identified using Scopus and Google Scholar, as well as the reference lists from existing sources, including the review by Hämäläinen et al. (2013). The keywords were “(auditory AND (frequenc* W/3 discriminat*) AND (dyslex* OR read* OR ‘read* difficult*' OR literac*))” for Scopus (where “W/3” indicates a word proximity search); and “(frequency AND discrimination) AND (dyslexia OR reading difficulties OR literacy)” for Google Scholar. Preliminary sorting of the resulting literature yielded 32 journal articles that examined frequency discrimination for pure tones in samples of developmental dyslexia, using psychophysical procedures with forced‐choice behavioural paradigms. Six studies were excluded due to: the use of an unselected (i.e., not dyslexia) sample; the inability to contact the authors or to obtain additional information required for the meta‐analyses; the replication of a participant group in a study that was already included in our nominal dataset; or frequency discrimination scores not reported as a threshold. This left 26 papers; those by Papadopoulos, Georgiou, and Parrila (2012), Banai and Ahissar (2006) and Goswami, Gerson, and Astruc (2010) contained multiple, independent estimates of effect size. Table 1 details the 30 effect size estimates used in the final analysis from the included studies.

**Table 1:** Studies included in the meta‐analysis, presented in order of increasing magnitude of effect‐size (Hedges *g*); and related descriptive statistics

		*g*	*SE*	Variance	Lower, upper limit	*Z*	*p*
1	Papadopoulos et al. (2012)^a^	−0.27	0.31	0.10	−0.88, 0.34	−0.86	.39
2	Santurette et al. (2010)	0.18	0.25	0.06	−0.31, 0.68	0.74	.46
3	Wijnen, Kappers, Vlutters, and Winkel (2012)	0.35	0.24	0.06	−0.11, 0.81	1.51	.13
4	Papadopoulos et al. (2012)^b^	0.36	0.26	0.07	−0.16, 0.87	1.35	.18
5	Georgiou, Protopapas, Papadopoulos, Skaloumbakas, and Parrila (2010)	0.37	0.30	0.09	−0.22, 0.96	1.22	.22
6	Walker, Shinn, Cranford, Givens, and Holbert (2002)	0.50	0.38	0.15	−0.25, 1.25	1.3	.19
7	Papadopoulos et al. (2012)^c^	0.53	0.33	0.11	−0.12, 1.18	1.60	.11
8	Amitay, Ben‐Yehudah, Banai, and Ahissar (2002)	0.54	0.28	0.08	−0.02, 1.10	1.90	.06
9	Watson and Miller (1993)	0.56	0.25	0.06	0.07, 1.10	2.26	.024
10	Banai and Ahissar (2004)	0.60	0.20	0.04	0.21, 1.00	3.02	.002
11	Thomson and Goswami (2008)	0.63	0.29	0.08	0.06, 1.20	2.17	.03
12	Ahissar, Lubin, Putter‐Katz, and Banai (2006)	0.65	0.33	0.11	0.00, 1.29	1.97	.049
13	Amitay, Ben‐Yehudah, et al. (2002)	0.65	0.23	0.05	0.20, 1.10	2.83	.005
14	Heath et al. (2006)	0.68	0.22	0.05	0.25, 1.10	3.11	.002
15	Hill, Bailey, Griffiths, and Snowling (1999)	0.68	0.35	0.12	−0.01, 1.37	1.94	.052
16	Ahissar et al. (2000)	0.70	0.34	0.11	0.04, 1.36	2.07	.038
17	Banai and Ahissar (2006)^d^	0.74	0.28	0.08	0.19, 1.28	2.66	.008
18	Gibson, Hogben, and Fletcher (2006)	0.76	0.22	0.05	0.33, 1.19	3.48	<.001
29	Halliday and Bishop (2006)	0.77	0.24	0.06	0.30, 1.23	3.24	.001
30	Banai and Ahissar (2006)^e^	0.82	0.32	0.10	0.19, 1.45	2.55	.011
21	Oganian and Ahissar (2012)	0.82	0.18	0.03	0.48, 1.17	4.69	<.001
22	Ben‐Yehudah and Ahissar (2004)	0.84	0.25	0.06	0.35, 1.34	3.33	.001
23	Wang, Huss, Hamailainen, and Goswami (2012)	0.85	0.21	0.05	0.43, 1.27	3.95	<.001
24	McArthur, Ellis, Atkinson, and Coltheart (2008)	0.96	0.21	0.05	0.54, 1.38	4.50	<.001
25	Goswami et al. (2010)^f^	1.01	0.28	0.08	0.46, 1.56	3.57	<.001
26	McAnally and Stein (1996)	1.04	0.3	0.09	0.45, 1.63	3.47	.001
27	Ben‐Yehudah, Banai, & Ahissar (2004)	1.16	0.30	0.09	0.57, 1.74	3.87	<.001
28	Goswami et al. (2010)^g^	2.12	0.3	0.09	1.53, 2.71	7.06	<.001
29	Hari et al. (1999)	2.45	0.46	0.21	1.55, 3.35	5.32	<0.001
30	Cacace, McFarland, Ouimet, Schrieber, and Marro (2000)	3.03	0.79	0.62	1.48, 4.57	3.83	<.001
Mean		0.76	0.80	0.01	0.60, 0.91	9.49	<.001

*Notes:* See Section 3 for exclusion criteria; three papers are included in the table more than once, because they contained more than one sample. Results are rounded to two decimal places; alpha values (*p*) to three decimal places.

a Grade 6 sample.

b Grade 4 sample.

c Grade 2 sample.

d 7th grade sample.

e 8th grade sample.

f Chinese language sample.

g English language sample.

The published manuscript from each study was examined to extract descriptive statistics for both the dyslexic and control groups on the constituent frequency discrimination task, as well as for additional psychometric variables. Those held in common by at least a subset of the studies (see Table 2), included non‐word and single word reading, phoneme deletion, Spoonerisms, non‐verbal IQ, and verbal digit span. We also extracted data regarding age and psychophysical task design from each study for use in moderator variable analyses.

**Table 2:** Measured variables used in the moderator analysis, including the number of studies that included reports of these measures

	Psychological construct	Measures	*n*
Standardised measures	*Non‐word reading*	TOWRE, WRMT‐R, Castles and Coltheart's Non‐word list	8
	*Real word reading*	TOWRE, WRMT‐R, WRAT‐III, Castles and Coltheart's irregular word list	8
	*Non‐verbal IQ*	Block design from WAIS‐III, WAIS‐R, WISC‐R95,WISC‐III; matrices reasoning from WAIS; MAT; KBIT matrices	19
	*Digit span*	CTPP, WAIS‐III, WAIS‐R, WISC‐R95	13
	*Verbal abilities*	Receptive vocabulary from ROWPVT; PPVT‐III; CELF‐III, BPVS; BPVS‐2; expressive vocabulary from WISC‐R95, WAIS‐III, WAIS‐R, WISC‐III; similarities from WAIS‐III;	16
Non‐standardised measures	*Non‐word reading*	Not specified by authors or not a published test	13
	*Real word reading*		10
	*Phoneme deletion*		8
	*Spoonerisms*		8

Abbreviations: BPVS, British Picture Vocabulary Scale; BPVS‐2, British Picture Vocabulary Scale – 2nd Edition; CELF‐III, Clinical Evaluation of Language Fundamentals; CTPP, Comprehensive Test of Phonological Processing; KBIT, Kaufman Brief Intelligence Test; MAT, Matrix Analogies Test – Expanded Form; PPVT‐III, Peabody Picture Vocabulary Test‐III; ROWPVT, Receptive subtest of the One Word Picture Vocabulary Test; TOWRE, Test of Word Reading Efficiency; WAIS‐R, Wechsler Adult Intelligence Scale – Revised; WAIS‐III, Wechsler Adult Intelligence Scale; WISC‐R95, Wechsler Intelligence Scale for Children; WISC‐III, Wechsler Intelligence Scale for Children – Revised; WRAT‐III, Wide Range Achievement Test; WRMT‐R, Woodcock Reading Mastery Tests‐Revised.

In a small minority of studies, descriptive data were not available from the manuscript at the level of precision required for meta‐analysis. In these cases, data were extracted from the published figures using the Data Thief III software (Tummers, 2006), where possible. Other studies had partitioned the dyslexic and control groups into subgroups according to a criterion variable (for e.g., frequency discrimination threshold or IQ score) and correspondingly reported descriptive statistics based on these subsamples. In these instances, we recalculated the means and standard deviations for the entire group based on the aggregate data. If the same dyslexic and control participants took part in more than one experimental condition, the mean effect‐size across all relevant conditions was used, with variance adjusted to account for repeated measures. When there were independent participant groups in the same paper, their results contributed to separate estimates of effect size.

### 3.2 Meta‐analysis of auditory frequency discrimination in dyslexia

The meta‐analysis, including the assessment of potential publication bias, was performed using the method described by Borenstein, Hedges, Higgins, and Rothstein (2009) and using commercial software (*Comprehensive Meta‐Analysis*, Biostat Inc.). Effect‐sizes for frequency discrimination were quantified using Hedge's *g*, with effect‐size heterogeneity estimated using a Q‐test. We employed a random‐effects model, which adopts the assumption that the constituent effect sizes have been sampled from a population distribution. Egger's test assessed the statistical significance of potential (non)publication biases, resulting from, for example, “file‐drawer” effects.

### 3.3 Post hoc analyses of moderator variables

Potential sources of variability in effect sizes across studies were explored with *post hoc* moderator variable analyses. Hypothesised effects of participant variables including age and cognitive skills were tested with simple meta‐regressions, each of which assessed associations of the between‐group effect‐sizes for psychometric and demographic variables with the effect‐size for frequency discrimination. Table 2 shows the composition of the moderator variables used in the meta‐regressions.

**Figure 1:**
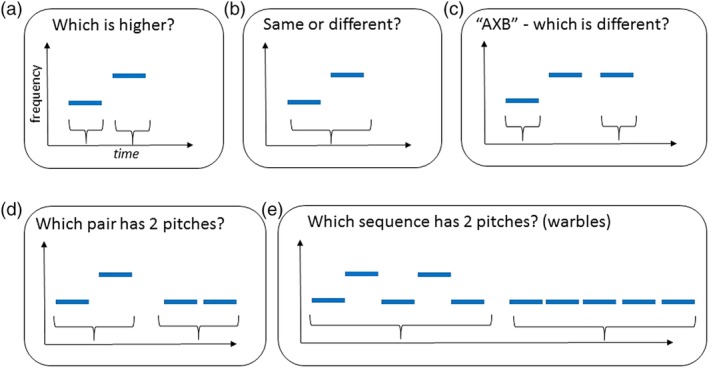
Illustration of the frequency discrimination task types used by the studies included in the meta‐analyses. For each task, the plot depicts the arrangement of tones over time and frequency, with brackets indicating the intervals, that is, response options, typically available to the participant. Typical task designs were as follows: (a) 2‐AFC tasks where participants were asked to identify which of two tones was higher in pitch. (b) Single‐interval tasks where participants were asked to report whether a pair of tones were the same or different in pitch. (c) AXB tasks involve the presentation of three tones, and this design was most typically used in 2‐AFC tasks (as illustrated), with one “reference” tone which never changed in the second position. Participants identified which of the other two tones flanking the reference differed in pitch. A similar 3‐tone design was also used in 3‐AFC tasks where any one of the three tones could differ. (d) 2‐AFC tasks involving two sequences of two tones, which were either identical or, in the target interval, had one tone with a different pitch. (e) Similar to task (d) but with a longer sequence of five tones in each interval

The effects of psychometric task variables were examined using non‐parametric analyses, due to the relatively small numbers of studies in each subsample. Figure 1 depicts the range of task types used in the sample of studies. Alternative types of task design yielded predictably different absolute frequency discrimination thresholds, due to variation both in the definition of criterion performance and because some tasks provided a greater number of exposures to a particular stimulus within a trial. This analysis relied on *effect‐sizes* obtained for between‐group comparisons, rather than the non‐standardised values associated with measures of absolute thresholds. When different task types were administered to the same participants within a study, each effect‐size was included in *post hoc* analyses, but when variants of the same task type were administered within a study, a composite effect‐size was used. Only the studies where the reference (standard) tone was constant across trials were used in the *post hoc* analysis; data from 3 conditions in 3 studies in Table 1 were removed from the analyses because they did not meet this criterion. One additional study was removed from the post‐hoc analysis of moderator variables because the frequency discrimination measurement was not reported as a threshold (Watson & Miller, 1993).

## 4 RESULTS

### 4.1 Meta‐analysis

**Figure 2:**
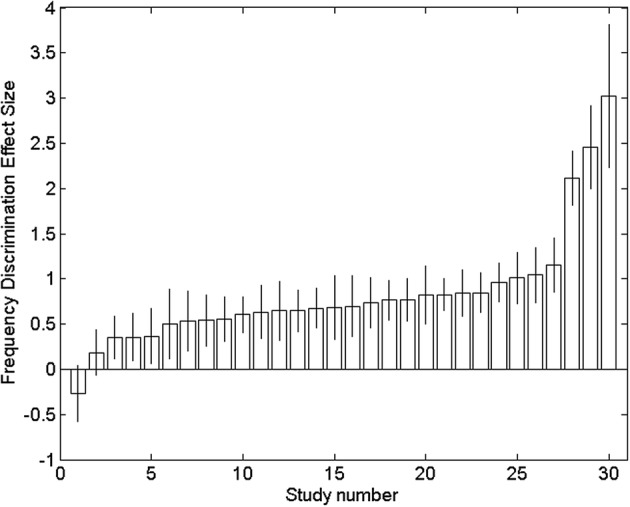
The Hedges' *g* and associated *SE* (±1) for each study, ranked and numbered as in Table 1

Figure 2 shows the ranked distribution of effect‐sizes and associated descriptive statistics for the individual studies listed in Table 1. The average effect‐size (*n*_effects_ = 30) was 0.76 (*SD* = 0.080; 95% CI: 0.60, 0.91). The *Q*‐test was significant (*Q* [29] = 73.6, *p* < .001), suggesting that the variance between studies differs significantly from zero. The between‐study variation as a proportion of the total variation (*I* ^2^) was estimated at 60.6% (95% CI: 41.3%, 73.5%). Egger's test was not significant, suggesting that although there is significant heterogeneity in effect‐sizes across studies, the results had not been systematically skewed, for example, by publication bias.

### 4.2 Analysis of moderator variables

**Figure 3:**
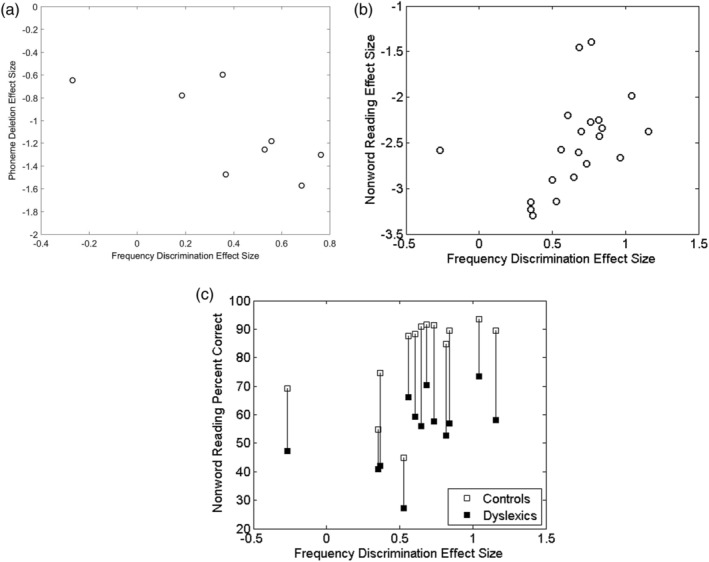
(a) Covariance between effect‐size for frequency discrimination and effect‐size for phoneme deletion (*n* = 8). Negative values for phonemic deletion indicate poorer ability on this measure for the dyslexia group compared to the controls. (b) Covariance between effect‐size for frequency discrimination and effect‐size for non‐word reading (*n* = 21). Negative values for non‐word reading indicate poorer ability on this measure by the dyslexia sample compared to controls. (c) Vertical lines reflect the difference in percent correct on non‐word reading between groups of dyslexics and controls from the same study

Table 3 summarises the main results obtained from the meta‐regressions between hypothesised moderator variables for participant characteristics across studies and the effect‐sizes for frequency discrimination. Two moderator variables predicted significant variance in effect‐size across studies: phoneme deletion and non‐word reading. Group differences in phoneme deletion skill – a construct that captures phonological language and short term and verbal working memory – yielded significant and moderately strong prediction of effect‐size for frequency discrimination (*β* = − 0.62, *p* = .03). As shown in Table 3 and depicted in Figure 3a, the slope (*β*) of this relationship is negative because higher individual frequency discrimination thresholds tend to represent *lower performance*. Thus, more positive effect‐sizes for phoneme deletion tasks were associated with more negative effect sizes for frequency discrimination, as predicted. Second, and more surprisingly, we identified a modest, yet statistically significant, *positive* relationship (*β* = 0.24, *p* = .04) between non‐word reading ability and the effect‐size for frequency discrimination. As shown in Figure 3b, the slope in this meta‐regression is positive, and *opposite* in direction to that which would be predicted by a hypothesised association between poorer frequency discrimination and lower phonological decoding skills. Here, effect‐sizes in non‐word reading that were less negative (i.e., smaller differences between groups) were associated with larger effect‐sizes for frequency discrimination. Although the magnitude of this relationship for phonological decoding is smaller than that obtained for phoneme deletion, similar marginally significant trends in this direction – which run opposite to that predicted – were also observed for non‐standardised measures of word and non‐word reading (but not for standardised measures of reading) (see Table 3). These effects may have resulted at least in part from the introduction of non‐standardised psychometric measures in some of the studies that contributed to the meta‐analysis. Alternatively, the differences in results across these phonological tasks represents the variability in the demands placed on other cognitive skills (for e.g., verbal memory) by the individual tasks.

**Table 3:** Results from the moderator variable analyses by meta‐regression

Moderator variable	*k*	Effect size		*I* ^2^(%)	Meta‐regression	
		*g*	95%CI		*β* (SE)	*p*
Age	19	0.74	[0.56, 0.92]	56.99	0.51 (0.3)	.09
NW stand.	8	0.73	[0.57, 0.89]	0	0.17 (0.17)	.31
NW non‐stand.	13	0.62	[0.44, 0.80]	27.55	0.30 (0.19)	.11
NW combined	21	0.67	[0.55, 0.79]	8.96	0.24 (0.12)	.04
RW stand.	8	0.70	[0.51, 0.88]	0	−0.11 (0.17)	.50
RW non‐stand.	10	0.59	[0.36, 0.82]	42.46	0.24 (0.14)	.08
RW combined	18	0.64	[0.50, 0.78]	17.20	0.14 (0.10)	.16
Phoneme deletion	8	0.41	[0.19, 0.63]	24.56	−0.62 (0.29)	.03
Spoonerisms	8	0.73	[0.55, 0.91]	0	0.33 (0.86)	.70
Non‐verbal IQ	19	0.68	[0.56, 0.80]	2.15	−0.14 (0.13)	.30
Digit span	13	0.71	[0.57, 0.84]	0	0.02 (0.35)	.96
Verbal abilities	16	0.64	[0.51, 0.77]	6.76	−0.08 (0.16)	.61

*Notes: k* = number of studies for a given moderator variable; *g* = the standardised group difference on FD for a given moderator variable (effect‐size); *I* ^2^ = proportion of the between‐study variation expressed as a percentage of the total the study variation; *β* = the relationship between the moderator and the frequency discrimination effect size; SE = standard error of *β*; *p* = alpha values for *β*; NW = nonword; RW = real word; stand. = standardised; non‐stand. = non‐standardised.

Different effect sizes for frequency discrimination were found for different task designs and were statistically significant across the sample of studies (Kruskall‐Wallis *H* = 11.2, *df* = 4, *p* = .024). Same‐different tasks as shown in Figure 1a had a mean effect‐size of 0.4 (*SD* = 0.27, *n* = 4); higher/lower tasks (Figure 1b) had a mean effect‐size of 0.92 (*SD* = 0.39; *n* = 9); and AXB tasks (Figure 1c) had a mean effect‐size of 1.10 (*SD* = 0.79, *n* = 11). Tasks asking participants to report which of two pairs differed in pitch, as illustrated in Figure 1d, yielded a mean effect‐size of 0.42 (*SD* = 0.40, *n* = 6); and the ten‐tone task depicted in Figure 1e, had an average effect‐size of 0.59 (*SD* = 0.13).

## 5 DISCUSSION

Our meta‐analytic review of the auditory frequency discrimination literature in dyslexia confirmed psychophysical evidence for deficits in samples of participants with developmental dyslexia compared to non‐impaired, control readers (see also Hämäläinen et al., 2013). The body of studies yielded overall group differences, with a mean effect‐size of the order of three‐quarters of a standard deviation. The population of studies was also characterised by significant inter‐study variability in the magnitude of these group differences, with our moderator variable analyses indicating that variables associated with both participants and tasks likely contributing to the heterogeneous pattern of result across studies.

A main aim of this study was to evaluate the extent to which impairment of auditory frequency discrimination is directly associated with phenotypic features linked with dyslexia rather than resulting from the mediating effects of third variables. We therefore used moderator variable analyses to explore relationships between frequency discrimination and reading‐component skills, non‐reading cognitive variables, and variability in the design of the psychophysical tasks.

The biological mechanism that underpins pitch perception, as required for frequency discrimination at low frequencies (up to 5 kHz) is phase‐locking; that is, the temporal association between neural firing and the stimulus waveform, that takes place in the auditory brainstem (e.g., Rose, Brugge, Anderson, & Hind, 1967; Schouten, 1970). Such low‐level sensory impairment of temporal processing could adversely impact the development of reliable and robust perception of pitch differences, and of pitch changes. However, some studies of auditory processing in dyslexia have used other stimuli (such as gap detection or detection of interaural time‐differences), the detection of which also depend on phase‐locking. These studies have provided mixed results of group differences, and overall the evidence for a generalised deficit in a basic, peripheral sensory impairment of phase locking in dyslexia is weak and inconsistent (Dougherty, Cynader, Bjornson, Edgell, & Giaschi, 1998; Edwards et al., 2004; McAnally & Stein, 1996; Patterson, Uppenkamp, Johnsrude, & Griffiths, 2002; Putter‐Katz, Feldman, & Hildesheimer, 2011; cf., Amitay, Ahissar, & Nelken, 2002; Chait et al., 2007; Johnson et al., 2013; Santurette et al., 2010). *Pitch perception* (cf. frequency encoding in brainstem neurons) is achieved through comparatively higher levels of processing in cortex. Pitch tuning has been demonstrated in individual neurons within auditory cortex (Wang & Walker, 2012), and functional neuroimaging evidence supports the existence of a cortical centre for the extraction of pitch (Griffiths & Hall, 2012). Perception of pitch therefore likely involves the contribution from a group of regions both within (Kumar & Schönwiesner, 2012) and beyond auditory cortex. Given the distributed nature of pitch processing, it is conceivable that an impairment which appears specific to frequency discrimination could also emerge from other processing differences in cortex, rather than at lower levels of the auditory processing hierarchy. Close interactions between sensory and relevant cognitive information processing in distributed cortical networks may therefore modulate the associations between frequency discrimination and reading that are observed in individual studies. This view is also consistent with alternative hypotheses, for example, that developmental dyslexia is associated more with problems in “perceptual anchoring,” used to establish the stimulus‐specific predictions necessary to accurately perform tasks like frequency discrimination, rather than with the presence of a primary sensory impairment (Ahissar, 2007).

### 5.1 Frequency discrimination and phonological awareness

Dyslexia has been strongly associated with deficits in phonological awareness (Stanovich, 1988; Wagner & Torgesen, 1987). The strongest predictive relationship between psychometric variables and effect‐sizes for frequency discrimination in the corpus of studies examined here was found for phoneme deletion, a task which taps explicit phonological awareness and verbal memory skills (see Table 3). This finding is based upon the results of subsample of studies (*n* = 7, 23%) that measured this construct. The strong covariance identified between frequency discrimination and such phonological awareness skills is consistent with several theoretical and experimental accounts of their mechanistic covariance (for e.g., Goswami, 2011; Talcott & Witton, 2002). Yet, the strong association between phoneme awareness and auditory frequency discrimination did not extend to all assessments of the phonological awareness construct. Comparatively weaker effects were found across studies which used other phonological tasks such as non‐word reading, and which place comparatively lower demands on short term memory processing and capacity. Table 3 shows that standardised measures of word and non‐word reading did not significantly co‐vary with frequency discrimination thresholds; the meta‐regression for the combined measure of non‐word reading yielded beta‐values with the opposite sign, that is, the relationship is in a direction opposite to that expected based on individual correlational studies. Figure 3c suggests that this finding may have been elicited primarily through the results of four studies – those depicted in the lower left quadrant of the plot. All of these studies employed a particular variant of the frequency discrimination task, in which multiple reference tones were presented (3 samples from Papadopoulos et al., 2012 and Georgiou et al., 2010), thereby placing increased demands on short term memory. Removing these studies from the analysis reduces the *β* value and correspondingly reduces the strength and significance value of the relationship between frequency discrimination and non‐word reading accuracy (*β* = 0.15, *p* = .28, *k* = 17). On the basis of these analyses there appears to be no strong evidence for a direct relationship between the effect size for frequency discrimination and that for either word or non‐word reading skill in the aggregate published literature.

A particular challenge that emerged in the moderator variable analyses was the inconsistency to which standardised psychometric measures for achievement and ability constructs were used in the constituent studies. For example, in less than a third of the studies were standardised measures of reading achievement reported, despite the central role of these measures to the conventional assessment of dyslexia. As a consequence of their bespoke psychometric properties and lack of normative data, therefore, the use of non‐standardised tests hinders the effective pooling of data across studies. An example of the potential impact of this issue is illustrated in Figure 3c, where mean scores for non‐standardised measures of non‐word reading (i.e., a subset of the studies in Figure 3b) are plotted against the effect‐sizes for frequency discrimination for each study. This plot illustrates how the impact of probable ceiling effects, particularly for the performance of the *control* participants in the study samples, may act to mask patterns of statistical relationships within the remaining data set. In this sample of studies, scores for the controls in the majority of studies ranged around 90% correct, and contrast with wider variability and lower mean scores for the dyslexic groups. These studies were characterised by their reliance on accuracy measures for measuring non‐word reading proficiency (10 of the 13 studies in Figure 3c), which both in adults and in transparent orthographies may yield ceiling effects on performance due to the paucity of errors committed by the average, typical reader. Hence, many of the non‐word reading tasks employed in the constituent studies may have been insufficiently sensitive to variability in the phonological skills of the control participants for use in providing reliable estimates of effect‐sizes for this variable.

### 5.2 Frequency discrimination and reading

Our meta‐regressions did not reveal the significant relationship between the effect sizes for real word reading and frequency discrimination that may have been predicted from a qualitative review of the literature. This finding also appears to run counter to the main outcome of the meta‐analysis, that is, that studies comparing dyslexic and control readers on tasks of frequency discrimination consistently find significant group differences. That the effect‐sizes for between group effects are larger than the comparative effect‐size correlation with continuous measures of performance on the core achievement variables upon which dyslexia is assessed, suggests that other cognitive variables likely account for significant variance in these between‐group differences. These hypothesized third variables may also include the compensatory effects of other cognitive and reading‐component skills that differ between groups.

### 5.3 Task design and other moderator variables

Moderator variable analysis of the impact of task design showed that psychophysical procedures with different designs tend to affect the magnitude of effect‐sizes for frequency discrimination, supporting the prediction that cognitive processes contribute at least in part to the inter‐subject variability in thresholds derived from this measure. However, in our meta‐regressions, we found that significant variance in frequency discrimination thresholds was neither accounted for by non‐verbal IQ (n = 19 studies), a composite verbal abilities measure (*n* = 17), nor verbal short term memory assessed psychometrically by digit span (*n* = 13 studies). This is despite observations from individual studies that performance on other auditory psychophysical tasks (for e.g., FM and AM detection, Witton et al., 2002) was statistically associated with individual differences in verbal short term memory capacity Many studies of dyslexia match participants at the group level for non‐verbal IQ, so is it is less surprising that this variable did not predict variance in frequency discrimination performance in our corpus of studies.

Other authors have attributed high within‐participant variability obtained on a frequency discrimination task to fluctuations in attentional allocation and control (Moore et al., 2008; Moore et al., 2010). In one study of normal adult listeners, 45% of the variance in performance on a frequency discrimination task was accounted for by attentional variables (Zhang et al., 2012). Our meta‐regressions were not able to fully test the impact of attentional variables on FD because most of the included studies did not contain relevant measures of attention. In recent modelling work, however we have shown how lapses in attention may significantly decrease the accuracy of threshold measurements in adaptive psychophysical procedures. In between groups comparisons, these differences in average lapse rates across groups – for example in dyslexia and control samples – increases the likelihood of Type 1 error in between‐group statistical contrasts (Witton et al., 2017). In a previous meta‐analysis of dyslexia, moderator variable analysis showed that between group effects in the literature on measures of postural control and balance were likely accounted for by a third variable other than reading – namely symptoms of ADHD, which were higher in the dyslexia group (Rochelle, 2006).

### 5.4 Heterogeneity and subtypes

A further factor related to the finding that cognitive variability may contribute to the heterogeneity of effect‐sizes for frequency discrimination is the broad behavioural variability inherent to the dyslexia phenotype. The genetic basis of dyslexia is understood to be both polygenic and heterogenic, and is linked to a number of candidate genes, each of which contributes to cumulative risk for developing dyslexia in association with other, non‐genetic factors (see Carrion‐Castillo, Franke, & Fisher, 2013, for review). Reading disability may therefore arise through one or more underlying mechanisms, which may vary across individuals, rather than being associated more with invariant, necessary conditions (Pennington, 2006). A corollary of such a multi‐mechanism approach to dyslexia that conceptualises causal risk factors as variable and multifactorial is the existence of subtypes of reading disability, based upon distinctive patterns across reading‐component skills (e.g., Castles & Coltheart, 1993) and amongst other underlying cognitive dimensions that constrain reading achievement (e.g., Bosse, Tainturier, & Valdois, 2007; Bowers & Wolf, 1993). Deficits on auditory frequency discrimination tasks may therefore only occur in particular clusters (i.e., subtypes) of poor reader, with distinct developmental trajectories. For example, Talcott et al. (2013) showed that deficits in frequency discrimination were statistically significant only in a group of children with poor phonological awareness skills. Differences in sampling the population of poor readers, whose trajectory of reading impairment may involve different cognitive mechanisms, may help explain the variability in effect‐sizes found across studies, particularly when inter‐ and intra‐group differences on underlying cognitive constructs interacts with performance on the dependent measure of interest.

## 6 CONCLUSION

Auditory frequency discrimination remains one of the more studied sensory abilities in samples of developmental dyslexia. Despite the large number of between group differences reported in the literature, the mechanisms that result in the group differences reported remain underspecified. Our meta‐analysis confirms overall group differences on this measure, despite significant variability in the strength of effects across studies. Variability in both participant factors and the design of the psychophysical tasks used for measuring frequency discrimination thresholds contribute to variability in effect‐sizes across studies. We suggest that previously underappreciated interactions between the nature of the tasks used to assess frequency discrimination and variability in the dyslexia cohorts on key cognitive dimensions is the key to understanding the importance of these relationships to the developmental phenotype of reading disability.

## Endnote

1 16 of the studies in this review used behavioural methods
